# The effectiveness of psychological interventions for reducing PTSD and psychological distress in first responders: A systematic review and meta-analysis

**DOI:** 10.1371/journal.pone.0272732

**Published:** 2022-08-24

**Authors:** Khalid M. Alshahrani, Judith Johnson, Arianna Prudenzi, Daryl B. O’Connor

**Affiliations:** 1 School of Psychology, University of Leeds, Leeds, United Kingdom; 2 Psychology Department, Faculty of Arts and Humanity, King Abdulaziz University, Jeddah, Saudi Arabia; 3 Bradford Institute for Health Research, Bradford Royal Infirmary, Temple Bank House, Duckworth Lane, Bradford, United Kingdom; 4 School of Public Health and Community Medicine, University of New South Wales, Sydney, Australia; 5 School of Psychology, University of Birmingham, Birmingham, United Kingdom; Rutland Regional Medical Center, UNITED STATES

## Abstract

**Background:**

First responders are faced with stressful and traumatic events in their work that may affect their psychological health. The current review examined the effectiveness of psychological interventions to treat posttraumatic stress disorder (PTSD), anxiety, depression, stress and burnout in first responders.

**Methods:**

Four databases were searched to identify controlled studies that examined the efficacy of psychological interventions to reduce PTSD symptoms (primary outcome) in first responders (including firefighters, police/law enforcement officers, search and rescue personnel, emergency and paramedics teams). Secondary outcomes were anxiety, depression, burnout, and stress.

**Results:**

15 studies were identified, including 10 studies that measured PTSD, 7 studies for anxiety, 10 studies for depression, 7 studies for stress and 1 for burnout. Interventions were associated with a significant reduction in PTSD (SDM = -0.86; 95% CI = -1.34 –- 0.39), depression (SDM = -0.63; 95% CI = -0.94 –-0.32), and anxiety (SDM = -0.38; 95% CI = -0.71 –-0.05) but not stress (SDM = -0.13; 95% CI = -0.51–0.25). CBT-based and clinician-delivered interventions were associated with significantly greater reductions in PTSD than other types of interventions and non-clinician interventions, but no differences were found for depression. There was evidence of moderate to high risk of bias across all studies.

**Conclusions:**

Psychological interventions are effective in reducing PTSD, depression and anxiety symptoms but not stress in first responders. Further research is needed using high quality randomised designs over longer periods of follow-up.

## Introduction

The term first responders has been used extensively in the literature to refer to a range of professions and occupations such as firefighters, police officers and paramedics [1–3]. These professions are considered by many as the ‘traditional’ first responders [4, 5]. However, there has been debate in the scientific and medical literature about which groups are classified as first responders [4]. For example, a number of studies have included 911 operators or dispatchers, correctional workers, nurses, physicians, public safety personnel and have drawn a distinction between ‘traditional’ and ‘untraditional’ first responders [4, 6]. Other studies have investigated risk workers which contain traditional and untraditional first responders, including public safety personnel and frontline healthcare professionals [7]. Nevertheless, recognising these definitional differences, the current research was interested in focussing on traditional first responders who respond to accidents or disasters in the early phases to protect and preserve life, property, and the environment; they include police officers, firefighters, search and rescue personnel, and emergency and paramedic teams [3].

Although the specific roles of different groups of first responders varies, they all face multiple potentially psychologically traumatic incidents in their daily work which puts them at heightened risk of experiencing mental health difficulties and disorders including posttraumatic stress disorder (PTSD), depression, anxiety and burnout [8–10]. In particular, first responders are more frequently exposed to potentially psychologically traumatic events than the general population, sometimes causing them to become ‘secondary traumatic victims’ [11–13]. Recent reviews have estimated the prevalence of PTSD and other psychological disorders among these populations. Petrie et al. [14] found prevalence rates in paramedics were 11% for PTSD, 15% for depression, 15% for anxiety, and 27% for general psychological distress. Other reviews have reported prevalence rates ranging between 6.4% to 57% for firefighters, and 5.8% to 19.6% for police officers [15–17]. As such, there is a pressing need to better understand the mental health of first responders and to identify efficient and appropriate interventions that are suitable for delivery to these groups. Due to the nature of their work, there is also a particular need to understand the effectiveness of interventions for helping to reduce PTSD symptoms in first responders.

In the last two decades, five systematic reviews have attempted to synthesize the effectiveness of interventions for reducing psychological distress among first responders, especially PTSD [2, 18–21]. Smith and Robert [18] identified a range of studies that tested interventions to reduce stress and PTSD among emergency ambulance personnel, and they found that all included articles (10 studies) had a lack of quality due to their methodological flaws such as, self-selection of groups and inadequate timing of the interventions. Haugen and his colleagues [2] reviewed papers that treated PTSD in first responders, and they found 17 articles but only two randomized controlled trials (RCTs). Alden et al. [19] evaluated the effectiveness of interventions targeting posttraumatic symptoms in first responders. They identified 21 studies and 8 of them were RCTs. They found that trauma focused therapies can be effective for first responders, but faced some limitations such as, the small sample sizes of most included studies, and few investigations with any control condition. Winders et al. [20] examined the prevention and treatment of psychiatric symptoms in first responders and how effectiveness was related to the income level of the country. They found, in 25 eligible studies, that most interventions were effective for preventing and treating psychological illness in first responders in all three levels of income countries. Another relevant review was conducted by Anderson et al. [2020] in frontline healthcare and public safety personnel. They reported inconsistent evidence regarding the effectiveness of organizational peer-support and crisis-focused psychological interventions for reducing PTSD symptoms but highlighted the need for further studies. However, a recent meta-analysis was published in 2021 [22] that examined the effectiveness of psychotherapy for posttraumatic stress injuries among public safety personnel. They found, by identifying 8 included studies, these interventions were associated with significant reductions in PTSD, anxiety, and depression symptoms.

Overall, three limitations are evident in the existing literature into interventions for psychological distress among first responders. First, controversy about the importance and effectiveness of interventions remains. While some studies and reviews have suggested that interventions are highly effective, others have reported no significant benefit of interventions [21, 23, 24]. Second, there appears to be a lack of high-quality intervention studies, and as such it is hard to draw firm conclusions based on the results of any study in isolation. Three, it is possible that some types of interventions are more effective than others, however, no studies or reviews have carefully investigated these potential differential effects. Cognitive Behaviour Therapy (CBT) and Eye Movement Desensitization and Reprocessing (EMDR) have been found to be effective in treating mental health disorders in both general clinical populations [11] and first responders [25], but there is a great deal of debate about the possible benefits of other interventions such as critical incident stress debriefing (CISD) [12]. In addition, previous meta-analyses have not compared the effectiveness of clinician delivered versus non-clinician delivered interventions or individual-based versus group-based interventions. In order to address these limitations, it was decided that a systematic review and meta-analysis were needed to assess the effectiveness of interventions used to treat PTSD and other aspects of mental health in first responders. The moderating effects of gender and age were also examined given that previous research has found that females and older workers have reported higher rates of PTSD compared to males and younger workers, respectively [26]. In addition, a number of important other methodological moderating factors were also explored (i.e., risk of bias, sample size, number of sessions and length of interventions).

## Aims and objectives

The first aim of the current review was to synthesise the evidence for the effectiveness of psychological interventions for improving mental health symptoms in first responders. Our primary outcome of interest was PTSD symptoms; secondary outcomes were depression, anxiety, stress, and burnout. Our second aim was to compare the effectiveness of different types and formats of interventions (CBT versus other interventions, clinician delivered versus non- clinician delivered interventions & individual-based versus group-based interventions) for reducing symptoms of PTSD, depression, anxiety, stress, and burnout.

## Method

This systematic review was registered in advance on PROSPERO, after specifying the inclusion criteria and analytical methods: CRD4MASKED. https://www.crd.york.ac.uk/prospero/display_record.php?ID=MASKED. The review followed the Preferred Reporting Items for Systematic Reviews and Meta-Analyses (PRISMA) guidelines that can be found in S1 Appendix.

### Search strategy

Four electronic databases were searched: EMBASE, PsycInfo, CINAHL, and Cochrane Register of Controlled Trials- from inception to June 2^nd^ 2019, updated to September 8^th^ 2021. The search strategy was performed by using three key blocks of terms: “interventions”, “first responders” and “posttraumatic stress disorder” or “depression” or “anxiety” or “burnout” or “stress”. Also, MeSH and keyword terms were examined based on terms usually used within previous reviews in the fields of first responders and PTSD (see eMethods.1 in S1 Appendix) [26–30].

### Eligibility criteria

Eligibility criteria were:

- Population: Studies that looked at first responders who work at the sites of critical incidents including police officers, firefighters, search and rescue personnel, and emergency and paramedic teams. We included any studies focused on these groups, including those with and without any pre-existing mental health diagnoses.
- Intervention: Studies with an individual or group psychological intervention designed to reduce PTSD, anxiety, depression, burnout, and/or stress symptoms that were delivered by registered clinicians (e.g., psychologists or psychiatrists) and non-clinicians (e.g., experiences police officers or supervisors).
- Comparison: Any type of control group (e.g., no intervention, alternative intervention, or wait list) was included.
- Outcomes: The primary outcome was PTSD symptoms as rated by an observer (e.g., doctor or researcher) using validated scales such as PTSD checklist [31] or self-reported PTSD symptoms measured using validated scales such as, PTSD symptoms scale self-report PSS-SR [32]. Secondary outcomes were stress, anxiety, depression, and burnout also measured via observer ratings or self-report, using validated scales (e.g., the Hospital Anxiety and Depression scales HADS [33], for anxiety and depression and the Maslach Burnout Inventory MBI, [34] for burnout).
- Design: Studies that used randomized controlled trial (RCTs) designs or controlled before-after designs (CBAs), as defined in the Cochrane handbook, were included [35].
- Context: Studies that investigated any treatment effects of psychological interventions in the first responders that were published in the English language were included.

### Exclusion criteria

We excluded studies conducted on other types of first responders, such as in-hospital health care workers (e.g., emergency room doctors and nurses) or military first responders (e.g., peacekeepers and soldiers). Also, all studies that used different types of interventions, such as physical or pharmacological interventions rather than psychological interventions were excluded, as were non-English language studies and gray literature.

### Study selection

All search results from each database were exported to Endnote version X8.2 (Clarivate Analytics, Philadelphia, United State) and all duplicates were removed. The study selection consisted of two stages. In the first stage, titles and abstracts were screened based on the above criteria by one author (KA). A second author (JJ) checked and reviewed 10% of included studies. To estimate the level of agreement we calculated the Kappa score which indicated good agreement (k = 0.845). In the second stage all full texts were screened by one reviewer (KA) with a second independent reviewer (AP) screening 20% of full texts, with 100% agreement.

### Data extraction

We used Excel 2016 [Microsoft Inc, Washington] to organize a data extraction form. We extracted quantitative data for the meta-analysis on a separate Excel file. All data extraction was undertaken by KA. The first 10% of eligible articles were independently extracted by AP to check for agreement. Any discrepancies were resolved by discussion. The following descriptive information was extracted from eligible studies:

- Study: country, research design, recruitment method and content of the control condition.
- Participants: sample size, age, gender, discipline, setting.
- Intervention: content of the intervention, measurement time points.
- Outcomes: PTSD, stress, anxiety, depression and burnout.

### Risk of bias (quality) assessment

We used the Effective Practice and Organisation of Care (EPOC) risk of bias tool [36] to carry out a critical assessment, as it is suitable for use across all different types of intervention designs, as reported in Cochrane handbook [35]. The tool comprises nine standardized criteria, each one rated on a three-point scale (0 = low risk, 1 = unclear risk, and 2 = high risk). Studies that obtained low-risk score have been considered across at least six of nine criteria to be less susceptible to risk of bias.

### Data analysis

Comprehensive Meta-Analysis software version 3 (CMA) [37] was used to calculate random effect sizes and other meta-analyses processes. The main meta-analysis examined the effectiveness of psychological interventions for improving PTSD in first responders. Secondary meta-analyses examined anxiety, depression, and stress interventions in same population.

We calculated the effect sizes using standardised difference of means (SDM) and associated 95% confidence intervals for PTSD, anxiety, depression, and stress outcomes in all included studies. We used post-treatment means, standard deviations (SD), and sample sizes to calculate the effect sizes between treatments and control groups. When means and SD values were not provided in the original studies, we used the available sample sizes and p-values to impute missing SDM (following the procedures outlined by Borenstein and colleagues (see Borenstein & Rothstein, 2013). For studies that had two types of interventions compared to a waiting list control group, we divided the sample size of the control group in half in each type to reduce the variance related with each effect size and to avoid double counting of participants [38]. If studies collected more than one follow-up assessment point, we used the first assessment point following the intervention.

For comparison groups, we identified the most common type of intervention used for each outcome and compared with all other interventions. If there was no common intervention for an outcome, then the outcome was not included in subsequent analyses. In addition, we also compared between interventions according to whether they were provided by clinicians versus non-clinicians (providers) and whether they were group-based versus individual-based interventions (format).

To account for heterogeneity, a random effects model was used in all analyses and assessed with I^**2**^ statistic [35]. I^**2**^ values indicate 25% as low, 50% as moderate, and 75% as high heterogeneity. In addition, between-study heterogeneity was assessed by Cochran’s Q-statistic (measure of weighted squared deviations) [39]. The Cochran Q test (significance level, *p* < 0.05) was used to compare between types of interventions. Specifically, the Q_between_ statistic was used to examine if the SDM was significantly different between groups of interventions. Moreover, meta-regressions were used to examine the impact of any potential moderating variables on the overall effect size for different outcomes. Meta-regression follows similar principles to regression or multiple regression in primary studies, except that in meta-analyses the variables are at the level of the study and not the participant [38]. Six moderating variables were investigated (risk of bias, gender, mean age, sample size, number of sessions, and total length of interventions by minutes). Sensitivity analyses were performed to: 1) examine the balance of the results among studies with a lower risk of bias rating and 2) to investigate the potential impact of outliers. Publication bias was calculated with Egger’s regression test [39], and further explored using funnel plots and Duval and Tweedie’s trim and fill analysis [40].

## Results

**Fig 1:**
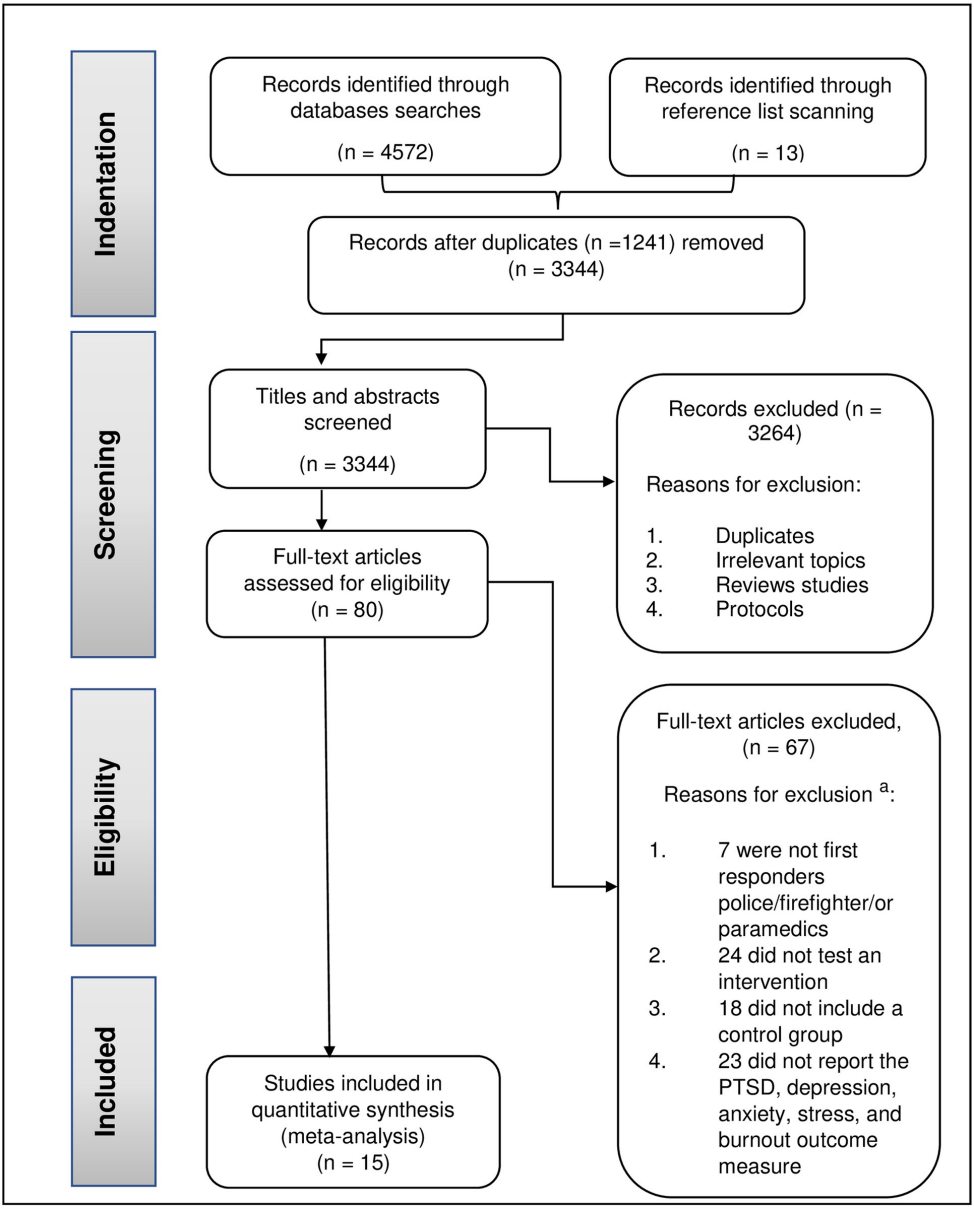
Flow diagram of article selection. ^a^Some studies were excluded for more than one reason so the total studies do not equal 66.

A total of 4572 papers were identified by our searches. In addition, 13 papers were discovered by manual reference list scanning (see Fig 1). We removed duplicated articles, and then screened the title and abstracts of 3344 studies. 80 out of these were screened as full text. 20 papers were eligible to include in the systematic review and meta-analysis [25, 41–58]. For articles which were eligible for the systematic review but which did not report the necessary outcome data for meta-analysis, we contacted the authors and co-authors, to request the relevant data. Five papers [25, 42, 46, 47, 59] were not included in the meta-analysis because either the authors no longer had access to the data or the authors did not respond. The total number of studies included was 15 [41, 44, 45, 50–56, 58, 59].

### Characteristics of the studies and participants

Across the 15 papers, the total sample size was 928 (mean age = 39.5 years, 64.8% males). 10 studies measured PTSD, 7 studies measured anxiety, 10 studies measured depression, and 7 studies assessed stress. One study evaluated burnout (this study was not meta-analysed due to there being an insufficient number of studies for meta-analysis). In terms of participants, 6 studies were conducted with police officers, 3 studies were conducted with firefighters, 2 studies were conducted with ambulance personnel, and 4 studies were conducted in a mix of disaster workers that included police officers, firefighters, and paramedics. 4 out of 15 studies were conducted in the USA, 3 in Australia, 3 in the Netherlands and one study each in England, Saudi Arabia, Iran, Thailand and Mexico.

### Characteristics of the interventions

A range of different types of interventions was used. Cognitive behaviour therapy (CBT) was the most frequent with 4 different types of CBT used in 3 studies, then critical incident stress debriefing (CISD) and resilience training were used in 3 studies each, while eye movement desensitization and reprocessing (EMDR) was used in 2 studies, and 1 study each used brief eclectic psychotherapy, eclectic group counselling, written emotional expression, and mental agility and psychological strength. One study had more than one intervention that used 2 types of cognitive behaviour therapy (the long type (CBT-L) and brief type (CBT-B)) compared to a waiting list control group [52]. The mean number of treatment sessions was 7.53, the longest individual session lasted 2.5 hours and the shortest one lasted 15 minutes (see Table 1). In terms of providers, two types of intervention deliverers were identified: clinician (8 studies) versus non-clinicians (6 studies), and one study [50] was not clear because there was no more details about providers or delivered type of interventions. In addition, interventions were delivered in an individual (8 studies) or group format (6 studies), and one study was not clear.

**Table 1:** Characteristics of studies, population and outcomes included in the review.

First author, year	Subjects & (Numbers (	Mean age	Gender	Country	Design	Intervention	Measurements	Control	Number of sessions	time points	Length of intervention by Minutes
Alghamdi, 2015	Firefighters (34)	30.4	Males	Saudi Arabia	RCT	NET (CBT)	SPTSS and HADS	Wait list	4 sessions (90 min per session)	Before, after treatment, 3 and 6 months follow up	360 minutes in all sessions
Behnammoghada, 2019	Emergency medical technician (50)	30.8	Unclear	Iran	RCT	EMDR	Alken stress	No intervention	5 consecutive sessions, each session lasting 45–90 minutes	Unclear	340 minutes in all sessions
Bryant, 2019	Police, firefighter, and paramedics (100)	43.6	Males = 77 Females = 23	Australia	RCT	CBT-L & CBT-B	CAPS & BDI	Wait list	12 sessions were 90 minutes.	Before, after treatment and 6 months follow up	1080 minutes in all CBT_L sessions
Carlier, 2000	Police officers (243)	31	Males = 173 Females = 70	Netherlands	CBA	CISD	SRS-PTSD & IES	No intervention	Three successive debriefing sessions	Before, after treatment, 6 months follow up	The mean of all was 74.7 minutes
Chongruksa, 2012	Police officers (26)	35.6	Unclear	Thailand	RCT	Eclectic group counseling	BDI-II and GHQ	Mental health psychoeducation	12 sessions each session lasting approximately 82 min	Before, after treatment and 1 month follow up	984 minutes in all sessions
Christopher, 2018	Police officers (61)	44	Males = 54 Females = 7	USA	RCT	MBRT	PROMIS measures (v1.0)	No intervention	8 sessions, each session spend 2 hours	Before, after treatment and 3 months follow up	1200 minutes in all sessions
Difede, 2007	Disaster workers (21)	45.7	Unclear	USA	RCT	CBT	PCL, BDI, and SCL-90	Treatment as usual	12 sessions each session lasting 75 minutes	Before, after treatment and 3 months follow up	900 minutes in all sessions
Gersons, 2000	Police officers (42)	36.5	Males = 37 Females = 5	Netherlands	RCT	BEP	HADS	Wait list	16 sessions, each session lasting 60 minutes	Before, after treatment and 3 months follow up	960 minutes in all sessions
Ireland, 2007	Police officers (67)	38.8	Males = 39 Females = 28	Netherlands	CBA	Written emotional expression	DASS	No intervention	12 work shifts for 15 minutes each day	No follow up	180 minutes in all sessions
Jarero, 2013	Paramedics and Firefighter (39)	Unclear	Males = 20 Females = 19	Mexico	RCT	EMDR	SPRINT	counseling group	2 sessions, each session lasting 90 minutes	Before, after treatment, 1 and 3 months follow up	180 minutes in all sessions
Ramey, 2016	Police officers (38)	41.2	Males = 29 Females = 9	USA	CBA	Resilience training	IES & PSS	Wait list	Two sessions, each session lasting 7.5 h	Unclear	900 minutes in all sessions
Skeffington, 2016	Firefighters (75)	28.8	Males = 71 Females = 4	Australia	RCT	The Mental Agility and Psychological Strength	PCL-C and DASS	Received treatment as usual	4 sessions, each one lasting 1 hour	Before, after treatment, 6 and 12 months follow up	240 minutes in all sessions
Tuckey, 2014	Firefighters (67)	Unclear	Males = 61 Females = 6	Australia	RCT	CISD	IES & K-10	No intervention	3 sessions which lasting 90 minutes	No follow up. Only pre and post treatment after 1-momth	270 minutes in all sessions
Wee, 1999	Emergency medical personnel (65)	Unclear	Unclear	USA	RCT	CISD	FRI-A	No intervention	12 days with no more details	Unclear	NA
Wild, 2020	Police, ambulance, firefighter, and search and rescue (430)	41	Males = 180 Females = 250	England	RCT	Resilience intervention	PCL-5, PHQ-9, and GAD-7	Psychoeducation	6 sessions each one lasting 150 minutes	Before, after treatment with 3 months follow up	900 minutes in all sessions

confidence intervals

**Abbreviations**: **NA**, indicates not available; **RCT**, Randomized Controlled Trials; **NET**, Narrative Exposure Therapy; **SPTSS**, Scale of Posttraumatic Stress Symptoms; **HADS**, Hospital Anxiety and Depression Scale; **CBA**, Controlled before-after study; **IES**, Impact of Events Scale; **BEP**, Brief Eclectic Psychotherapy; **CAPS**, Clinician Administered Posttraumatic stress disorders Scale; **BDI**, Beck Depression Inventory; **CISD**, Critical Incident Stress Debriefing; **SRS-PTSD**, Self-Rating Scale for Posttraumatic stress disorders; **MBRT**, Mindfulness-Based Resilience Training; **BDI-II**, Beck Depression Inventory-Second edition; **GHQ**, General Health Questionnaire; **CBT**, Cognitive behavioural Therapy; **PCL**, PTSD Checklist; **PHQ-9**, Patient Health Questionnaire; **GAD**, Generalised Anxiety Disorder questionnaire; **SCL-90**, Symptom Checklist 90; **DASS**, Depression Anxiety Stress Scales; **PSS**, Perceived Stress Scale; **K-10**, Kessler-10; **FRI-A**, Frederick Reaction Index-Adult; **PTDS**, Posttraumatic Stress Diagnostic Scale; **PSI**, Police Stress Inventory; **JSS**, Job Stress Survey; **EMDR**, Eye Movement Desensitisation Reprocessing.

### Risk of bias characteristics

The risk of bias was calculated using the EPOC 9 criteria. 8 studies were considered low risk, 6 studies were scored as moderate risk, and 1 study was high risk (see eFigure 1 in the S1 Appendix). In addition, in 6 of the studies the comparison or control condition did not include any intervention component. Future research should endeavour to include an active control condition. Lack of blinding across studies was also a potential source of high risk of bias given that many studies did not use blinding or it was not reported clearly in the papers. Similarly, there were some concerns related to lack of allocation concealment and missing data.

### Main and secondary meta-analysis

**Fig 2:**
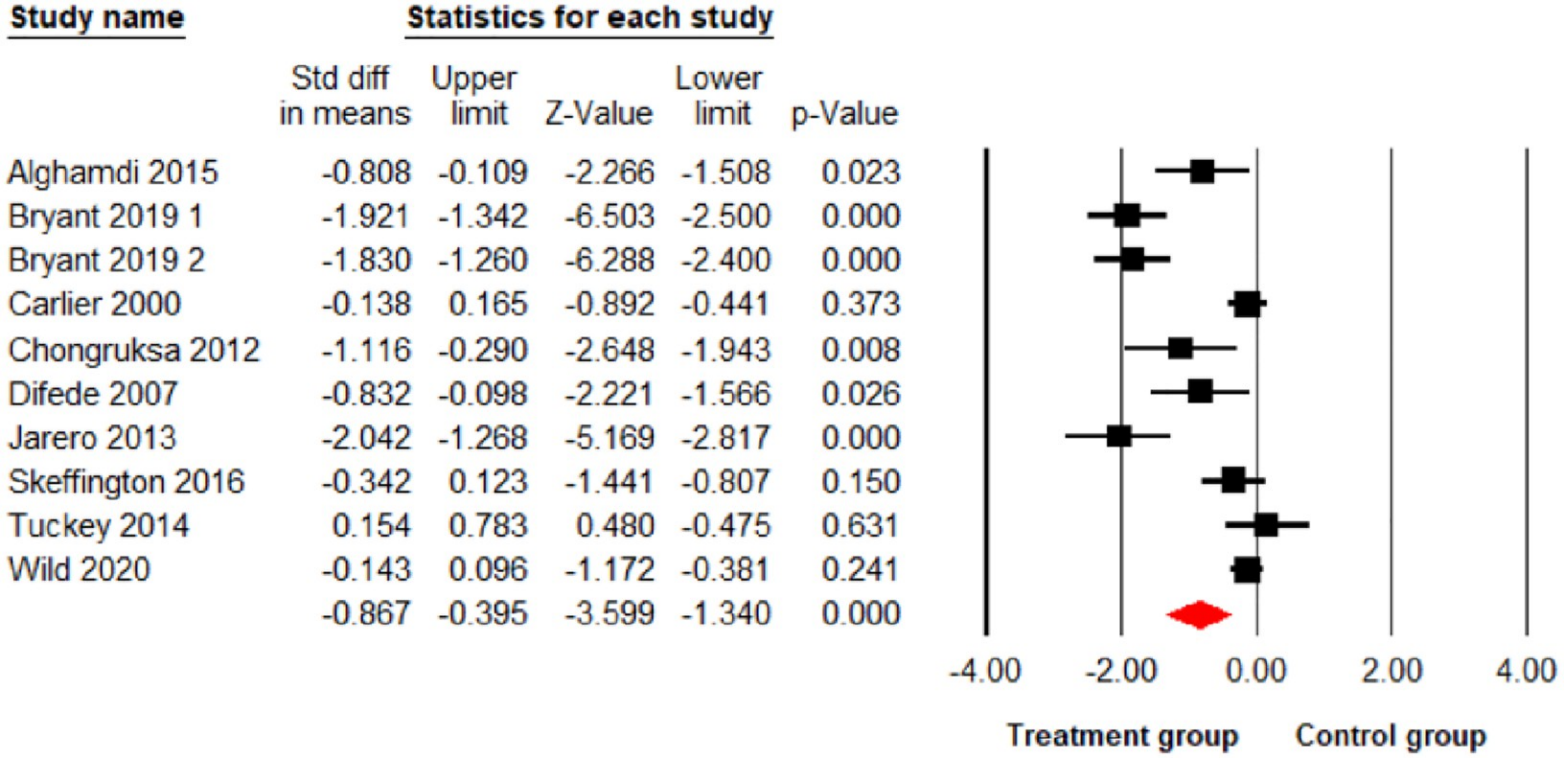
Forest plot for the effect of interventions on PTSD symptoms.

**Fig 3:**
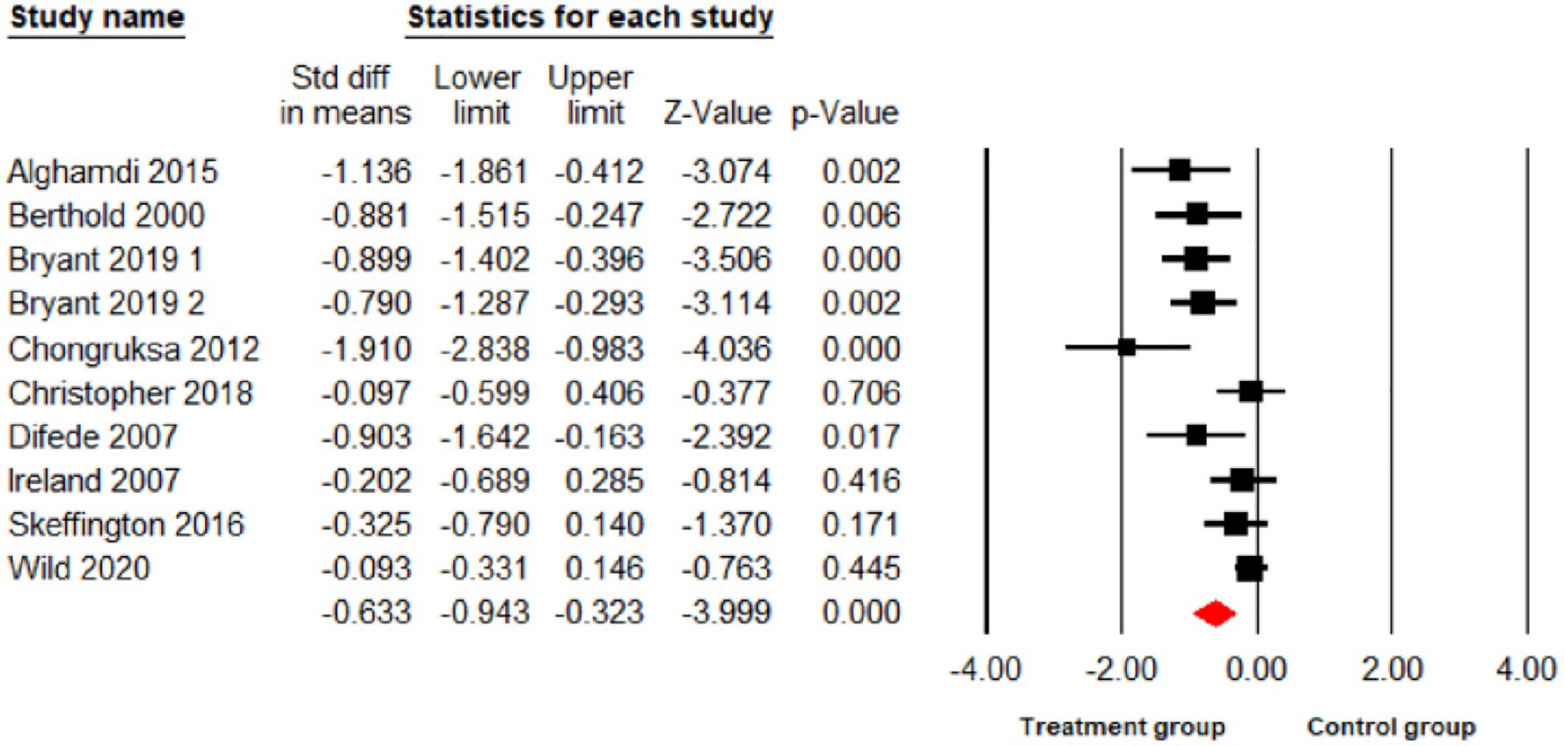
Forest plot for the effect of interventions on depression symptoms.

**Fig 4:**
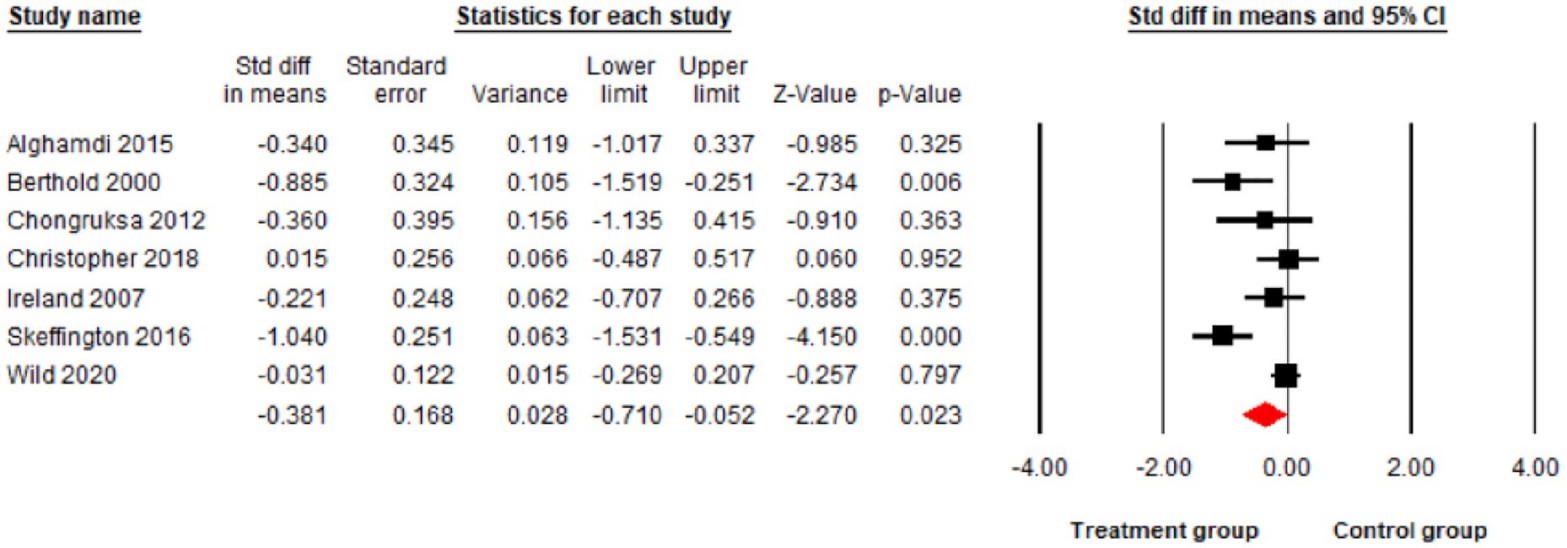
Forest plot for the effect of interventions on anxiety symptoms.

**Fig 5:**
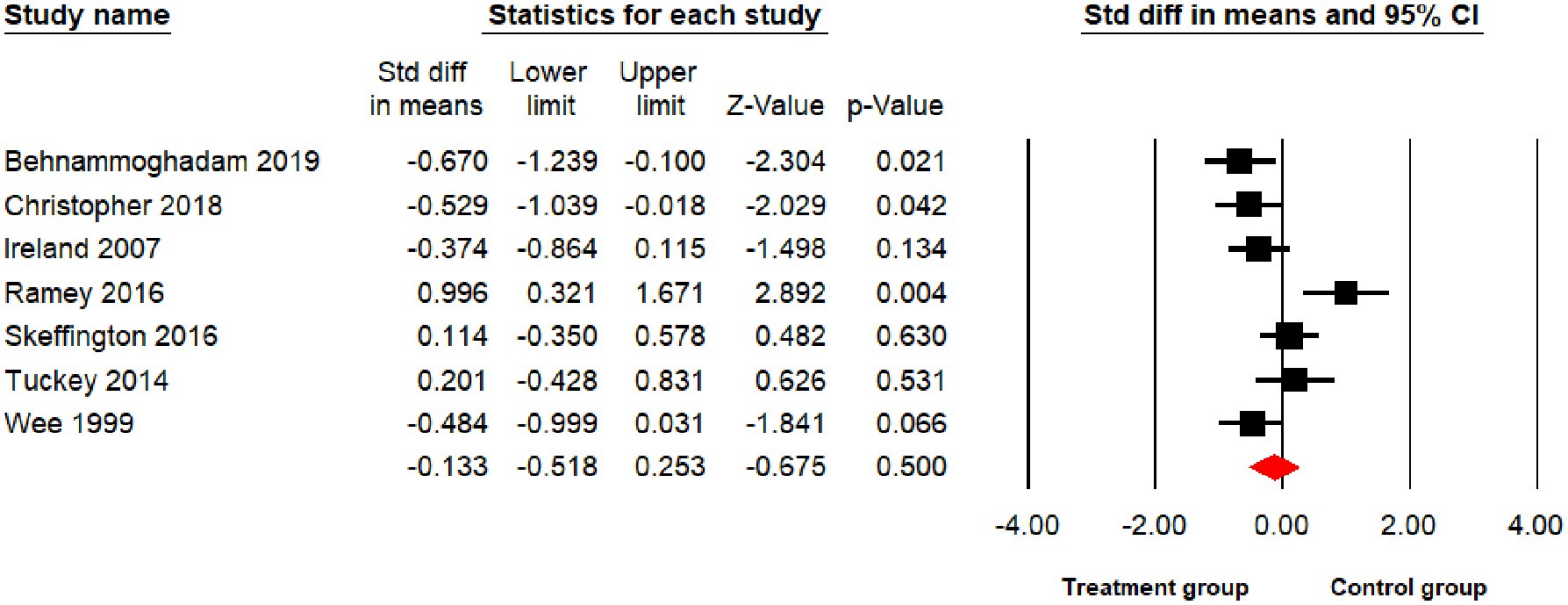
Forest plot for the effect of interventions on stress symptoms.

The main meta-analysis results found psychological interventions led to a significant reduction in PTSD and depression at p<0.01 level (10 comparisons: SDM = -0.86; 95% CI = -1.34 –-0.39; I^2^ = 88.97%; Fig 2), (10 comparisons: SDM = -0.63; 95% CI = -0.94 –-0.32; I^2^ = 72.48%; Fig 3) respectively, and a significant reduction in anxiety symptoms at the p<0.05 level (7 comparisons: SDM = -0.38; 95% CI = -0.71–0.05; I^2^ = 67.12%; Fig 4) but no significant reduction for stress (7 comparisons: SDM = -0.13; 95% CI = -0.51–0.25; I^2^ = 71.67%; Fig 5). As I^2^ values shown above, the heterogeneity between the studies in the PTSD analysis was high indicating the need to consider the role of potential moderators. Therefore, the following 6 moderators were considered: risk of bias, gender, mean age, sample size, session number and the length of treatments (See Table 3).

### Comparing between interventions

Cognitive behaviour therapy was the most common intervention (4 comparisons from 3 studies) used to treat PTSD, anxiety and depression. However, across the 7 anxiety studies, CBT was used only once, therefore, we were only able to compare CBT with all other interventions for the outcomes of PTSD and depression. We also compared interventions delivered by clinicians (8 studies) against those delivered by non-clinicians (6 studies) and the interventions that were delivered individually (8 studies) versus those provided in a group format (6 studies) for PTSD and depression outcomes. In terms of comparing between CBT and other interventions, the results showed that there were statistically significant differences between the standardised difference in means between the CBT and all other interventions for PTSD symptoms, such that CBT was more effective (Q = 5.74; *p* = 0.01), but this was not found for depression, where the difference was non-significant (Q = 3.44; *p* = 0.06). Interventions delivered by clinicians were associated with greater reductions in PTSD compared with those delivered by non-clinicians (Q = 7.59; *p*< 0.001), but interventions delivered individually were not associated with greater reductions in PTSD than group interventions (Q = 3.47; *p* = 0.06). For depression, however, there were no differences between clinician compared with non-clinician delivery (Q = 1.78; *p* = 0.18), or individual compared with group format (Q = 2.46; *p* = 0.11). See Table 2.

**Table 2:** Comparing between three categories of interventions: CBT versus other interventions; clinician versus non-clinician providers; and individual versus group interventions for PTSD and depression.

Outcome	Comparator	N	SDM (p)	CIs	I^2^	Q (p) within studies	Q (df) & p between the groups
PTSD	CBT	4	-1.38 (0.00)	-0.81; -1.95	70.58	10.19 (0.017)	5.742 (1) 0.01
	Other interventions	6	-0.50 (0.02)	-0.06; -0,94	82.02	27.81 (0.00)	
Depression	CBT	4	-0.90 (0.00)	-1.19; -0.60	0.000	0.59 (0.89)	3.449 (1) 0.06
	Other interventions	6	-0.41 (0.01)	-0.68; -0.18	72.62	18.26(0.00)	
PTSD	Clinician	7	-1.20 (0.00)	-1.48; -0.97	82.93	35.15 (0.00)	7.59 (1) <0.001
	Non-clinician	3	-0.20 (0.00)	-0.45; 0.06	0.000	0.61(0.73)	
Depression	Clinician	6	-0.77 (0.00)	-0.97; -0.50	24.70	6.64 (0.25)	1.78 (1) 0.18
	Non-clinician	4	-0.39 (0.06)	-0.40; -0.01	78.96	14.26 (0.00)	
PTSD	Individual	6	-1.23 (0.00)	-1.07; -0.65	90.88	54.83 (0.00)	3.47 (1) 0.06
	Group	4	-0.33 (0.37)	-0.39; -0.00	53.98	6.51 (0.089)	
Depression	Individual	6	-0.77 (0.00)	-0.97; -0.50	24.70	6.64 (0.25)	1.78 (1) 0.18
	Group	4	-0.39 (0.06)	-0.40; -0.01	78.96	14.26 (0.00)	

Note * **N** = number of comparison; **SDM** = Standard difference of means; **CI** = Confidence interval; **I**^**2**^ = score of heterogeneity; **Q** = Cochrane’s Q test statistic testing for between group differences

### Tests of moderation

The moderate risk (n = 6) and high risk (n = 1) studies were combined and compared with the low risk (n = 8) studies using meta-regression. The results showed that there was moderating effect of risk of bias for only PTSD (Q = 5.05; *p* = 0.01), such that studies with low risk was reported to have higher SDMs. However, there were no moderating effect of risk of bias for anxiety (Q = 0.59; *p* = 0.44), depression (Q = 1.13; *p* = 0.28) and stress outcomes (Q = 0.41; *p* = 0.81) (see Table 3). We also examined the moderating effects of gender, mean age, sample size, number of intervention sessions and the total number of minutes of interventions (Table 3). For PTSD, only the number of sessions was found to be significant moderators (Q = 14.6; *p* = 0.001), such that studies with more intervention sessions was found to have larger SDMs. For anxiety, only mean age was found to be a significant moderator on the outcome, such that studies with a higher mean age had a larger SDM (Q = 10.4; *p* = 0.01). For depression and stress there were no significant moderators. Moreover, gender was found to have no moderating effects on any of the outcomes.

**Table 3:** Meta-regression analyses relating to sample size, risk of bias, number of sessions, and the total length of intervention minutes.

Outcome	Moderator	B	R^2^	SE	Q	P
**PTSD**	Risk of bias	1.07	0.50	0.45	5.50	0.019
	Gender	0.00	0.39	0.00	3.40	0.065
	Mean age	-0.07	0.00	0.05	2.30	0.129
	Sample size	0.00	0.08	0.00	2.05	0.152
	Number of sessions	-0.18	0.73	0.04	14.6	0.001
**Anxiety**	Risk of bias	0.14	0.00	0.00	0.59	0.441
	Gender	0.00	0.00	0.83	0.00	0.973
	Mean age	0.06	0.93	0.02	10.4	0.001
	Sample size	0.00	0.00	0.00	1.95	0.328
	Number of sessions	-0.01	0.00	0.04	0.05	0.820
**Depression**	Risk of bias	0.29	0.18	0.27	1.13	0.287
	Gender	0.00	0.18	0.02	1.64	0.200
	Mean age	0.01	0.00	0.02	0.17	0.682
	Sample size	0.00	0.25	0.00	1.99	0.158
	Number of sessions	-0.03	0.19	0.03	0.97	0.325
**Stress**	Risk of bias	-0.48	0.00	0.61	0.41	0.813
	Gender	-0.01	0.00	0.22	0.41	0.521
	Mean age	-0.01	0.00	0.06	0.04	0.844
	Sample size	-0.02	0.04	0.02	1.79	0.181
	Number of sessions	-0.12	0.56	0.05	4.29	0.038

Note **B** = Beta result; **R**^**2**^ = Proportion of total between- study variance; **SE** = Standard Error; **Q** = Cochrane’s Q test statistic testing for between group differences; **P** = significance level

### Publication bias

Egger’s regression coefficients found evidence of publication bias in the depression studies (intercept = -3.97; 95% CI, -6.07 –-1.34; p = 0.003), but not for PTSD (intercept = -4.57; 95% CI, -8.64 –-0.51; p = -0.031), anxiety (intercept = -2.29; 95% CI, -6.14–1.55; p = 0.186) and stress (intercept = 7.36; 95% CI, -6.58–21.3; p = 0.230) (See e-figures 2–5 in S1 Appendix). However, a funnel plot suggested that missing studies were reported in only the right side of the mean for PTSD and depression (4 studies in each), and 3 studies in anxiety. Duval and Tweedie’s trim and fill analysis [40] found a small reduction in the SMD after missing studies for PTSD, depression, and anxiety were imputed (SDM = -0.21; CI, -0.34 –-0,08), (SDM = -0.26; 95% CI, -0.39 –-0.12), and (SDM = -0.07; CI, -0.22–0.07) respectively.

### Sensitivity analyses

When we used a leave-one-out method [35], the finding for PTSD and depression varied (between -0.73 and -0.98 for PTSD and between -0.53 and -0.71 for depression), but both outcomes remained significant, suggesting the findings were not driven by any single study. In contrast, there were two studies in the anxiety analysis [48, 60] which when removed in isolation led the analyses to become statistically non-significant (SDM = -0.304; p = 0.07) and (SDM = -0.214; p = 0.08) respectively. Similarly, there was one study in the stress analysis [45] which when removed led to a statistically significant finding (SDM = -0.133; p = 0.50).

## Discussion

The main aim of the present systematic review and meta-analysis was to synthesise the evidence about the effectiveness of psychological interventions for improving mental health symptoms in first responders. A secondary aim was to compare the effectiveness of different types and format of interventions (CBT versus other interventions, clinician delivered versus non- clinician delivered interventions & individual-based versus group-based interventions) included in the review. The results of the meta-analysis showed that psychological interventions were associated with a significant reduction in PTSD symptoms, depression, and anxiety, but not stress. Moreover, the analyses also revealed that CBT was significantly more effective than other interventions for PTSD only, not for depression symptoms. Interventions delivered by clinicians were found to be more effective for PTSD outcomes but not for depression outcomes.

Subgroup analyses also indicated that interventions were more effective in studies that were classified as having low risk of bias contrasted to the moderate-to-high risk of bias studies. The number of sessions was found to be a significant moderator for interventions for PTSD, such that more sessions were associated with greater intervention effectiveness. For anxiety outcomes, only the mean age was found to be a significant moderator of intervention effectiveness, such that higher mean age was associated with greater intervention effectiveness. For depression and stress, no significant moderating variables were found.

The main finding of this meta-analytic review is in line with most previous systematic reviews [2, 18–22] that psychological interventions are effective for reducing PTSD symptoms among first responders. Haugen et al. [2] found large significant treatment effects in two RCT designs included in their review. While Alden et al. [19] found, in 8 RCTs out of 21 studies included in their review, that psychological interventions were effective for treating PTSD symptoms in first responders. Similarly, with additional other types of first responders (e.g., military soldiers, nurses, and doctors), Winders et al. [20] found 13 out of 18 studies that evaluated psychological treatments reported a positive impact of interventions for preventing and treating psychiatric symptoms in first responders. Furthermore, in a review of studies in frontline healthcare and public safety personnel, Anderson et al. [21] found some mixed evidence for the effectiveness of organizational peer-support and crisis-focused psychological interventions. Similarly, Bahji et al. [22], in their meta-analysis found that the psychological interventions reduced PTSD symptoms in public safety personnel. Therefore, taken together, there is clear evidence from multiple reviews that psychological interventions are effective in helping to treat PTSD in first responders.

However, the current review differs in three important ways from previous reviews. Firstly, this is the first meta-analysis to compare between the effectiveness of intervention types. Secondly, unlike other reviews [2, 18, 21] our meta-analysis focused on only two types of study designs (RCTs and controlled before-after designs), thereby, providing more robust evidence of the effectiveness of psychological interventions in this context. Thirdly, our review covered a range of mental health outcomes in addition to PTSD.

There are several similarities between our current review and a recently published meta-analysis [22]. However, our present review differs to this in four key ways:(1) Bahji et al. [22] focused on ‘public safety personnel’, while this meta-analysis investigated first responders using the ‘traditional’ definition; (2) Bahji’s et al. [22] limited their searches from 2008 to 2019 only, while we searched from database inception to 2021, which explains the larger number of studies in this meta-analysis (15 studies compared with 8 studies); (3) Unlike [22], the present meta-analysis specifically compared between three different elements of interventions: CBT versus other interventions, clinicians versus non-clinicians, and individual versus group interventions; (4) Unlike [22], in the current meta-analysis, we also examined the effects of 6 moderators which were gender, mean age, sample size, number of intervention sessions and the total number of minutes of interventions by using meta-regression analysis.

Moreover, our review was the first to test whether there was any difference in the effectiveness of different types of psychological interventions in first responders, and the first to report that CBT was significantly more effective than other types of psychological interventions for reducing PTSD only, not for depression. This pattern of findings is consistent with findings from previous meta-analyses in non-first responder groups. Researchers have reported that several psychological treatments are effective for reducing PTSD in adults [54, 61]. For example, two meta-analytical reviews [62, 63] reported that trauma-focused cognitive therapy (TF-CBT) and EMDR were the most effective psychological therapies for treating PTSD in adults. While another recent meta-analysis [64] found that interpersonal psychotherapy was effective for reducing PTSD. However, most included studies in those meta-analyses were adults who were ‘first victims’ (e.g., sexual assaults, car accidents, refugees) who often suffered from one traumatic event. This is different to first responders who can be regarded as ‘second victims’, and who experience multiple traumas in their daily work. Therefore, the nature of their work makes them different from other populations due to the barriers they suffered such as stigma, fear of losing their job, and not knowing where to get help [65]. In terms of intervention providers, our findings suggested interventions delivered by clinicians were significantly more effective for reducing PTSD symptoms compared to those delivered by non-clinicians. However, there were no significant differences between individual versus group delivery or clinician versus non-clinician delivery for depression outcomes. Therefore, taken together, the current findings suggest, where possible, that future interventions targeting PTSD symptoms ought to be delivered by clinicians.

We also found that the number of sessions was a significant moderator in PTSD studies such that psychological interventions were more effective in studies with a higher number of treatment sessions. This finding is consistent with some previous reviews [66, 67]. Another significant moderator in studies with anxiety outcomes was age such that psychological interventions were more effective in studies with participants with higher mean age. This finding may be explained by the fact that symptoms of anxiety have been found to be higher in older compared to younger [68].

Surprisingly, the current systematic review and meta-analysis only identified a small number of RCT studies conducted in paramedics. It is now established that the prevalence of PTSD is higher in paramedics than other first responders. Therefore, there is an urgent need to investigate the effectiveness of psychological interventions in paramedics group. Yet, we only found 2 studies in paramedics compared with 6 in police officers, 3 in firefighters, and 4 in mixed disaster workers. Also, there is a clear lack of studies that treat burnout among the same population, and this is concerning as the burnout prevalence rate among paramedics has been found to range from 16% to 56% [69]. Therefore, our findings suggest that future research ought to conduct further, higher quality RCTs aimed at improving mental health outcomes in paramedics (as well as other first responders).

All included studies were from 8 different countries, and most of them (11 out of 15 studies) were from the USA, Australia, England, and the Netherlands, which limits the generalisability of the current findings. Differences between developing and developed countries in terms of prevalence of PTSD have been reported [70]. Also, there are differences in the type and availability of psychological interventions for PTSD and mental health generally in developed and developing countries. Therefore, it would be useful for future research to conduct comparative studies between these countries in terms of the types and frequency of traumatic events first responders are exposed to, the available supports they use and the effectiveness of any existing interventions.

### Strengths and limitations

The present systematic review and meta-analysis has a number of strengths. The design of this systematic review followed the guidance of the Cochrane handbook for systematic reviews of interventions [35] and the Preferred Reporting Items for Systematic Reviews and Meta-Analysis (PRISMA) [71]. The protocol of our review was registered on the PROSPERO database. Also, the current meta-analysis focused on RCT designs, which have numerous benefits such as providing strong empirical evidence of treatment’s efficacy, randomisation of the participants to the intervention and control groups and minimisation of the allocation bias [72].

Nevertheless, we are aware that there are also some limitations that ought to be acknowledged. The findings are based on a relatively small number of studies and there was a high level of heterogeneity between studies due to differences in the included studies, such as being conducted in developed versus developing countries, outcome measures, sample sizes, and length of interventions. Even though the review attempted to account for the heterogeneity by conducting subgroup analysis and meta-regressions, the variance was still high. Another limitation was that many of the studies have relatively short follow-up periods, therefore, we are unable to draw firm conclusions about the longer-term effects of interventions [73]. This is important here because first responders are vulnerable to multiple traumas in their work, even after receiving treatment, and follow-up assessment helps determine whether treatment gains can be maintained when exposed to various traumatic events [19]. Furthermore, overall, there were seven studies that were scored as having moderate or high risk of bias total scores, and six studies with no intervention in control groups that may lead to the blinding bias Therefore, future studies should ensure that participants are blinded, include active control conditions, make sure that missing data are addressed adequately and allocation concealment is utilised to help reduce risk of bias. Also, the publication bias analyses identified that there were some missing studies in the right-hand side of the funnel plots for PTSD, depression, and anxiety. A number of factors may account for this potential publication bias. For example, it might be because of the relatively small number of studies conducted in this area and/or that many of the studies are underpowered due to small sample sizes leading to small effects. Therefore, researchers should endeavour to prioritise these types of studies and ensure that future studies are adequately powered, preregistered and embrace open research practices as much as possible [74, 75]. It might also be fruitful for future research to explore the effectiveness of psychological interventions on other stress-related physiological outcomes (e.g., cortisol levels; cf., [76]), as well as psychological outcomes. We also recognise that we did not include other types of first responders (e.g., emergency room personnel; military personnel) and recommend that further research reviews whether interventions may help reduce mental health problems in these groups.

## Conclusion

The current systematic review and meta-analysis found that psychological interventions were effective for reducing PTSD, depression, and anxiety symptoms but not for stress in first responders. Further research is needed to estimate the effectiveness of other types of interventions with high quality RCT designs over longer periods of follow-up.

## Supporting information

S1 Appendix Supplemental digital. (DOCX)
