# Supplementary material for: The effectiveness of psychological interventions for reducing PTSD and psychological distress in first responders: A systematic review and meta-analysis
Source: PLoS One. 2022 Aug 24;17(8):e0272732. doi: 10.1371/journal.pone.0272732 (PMC9401173; doi:10.1371/journal.pone.0272732)
Supplement: S1 Appendix — (DOCX) [file pone.0272732.s001.docx]

**Supplemental Digital Appendix 1**

**Table of Contents**

| eTable 1. Completed PRISMA checklist | 2 |
| --- | --- |
| eMethods 2. Search strategy for EMBASE | 4 |
| eFigure 1. Ratings of studies included in the review on the 9 EPOC risk of bias criteria | 6 |
| eFigure 2. Funnel plot of standard differences of mean versus standard errors for depression | 7 |
| eFigure 3. Funnel plot of standard differences of mean versus standard errors for PTSD | 7 |
| eFigure 4. Funnel plot of standard differences of mean versus standard errors for anxiety | 8 |
| eFigure 5. Funnel plot of standard differences of mean versus standard errors for stress | 8 |
|  |  |

| **Section/topic** | **#** | **Checklist item** | **Reported on page #** |
| --- | --- | --- | --- |
| **TITLE** | | |  |
| Title | 1 | Identify the report as a systematic review, meta-analysis, or both. | 2 |
| **ABSTRACT** | | |  |
| Structured summary | 2 | Provide a structured summary including, as applicable: background; objectives; data sources; study eligibility criteria, participants, and interventions; study appraisal and synthesis methods; results; limitations; conclusions and implications of key findings; systematic review registration number. | 3 |
| **INTRODUCTION** | | |  |
| Rationale | 3 | Describe the rationale for the review in the context of what is already known. | 4 |
| Objectives | 4 | Provide an explicit statement of questions being addressed with reference to participants, interventions, comparisons, outcomes, and study design (PICOS). | 7 |
| **METHODS** | | |  |
| Protocol and registration | 5 | Indicate if a review protocol exists, if and where it can be accessed (e.g., Web address), and, if available, provide registration information including registration number. | 7 |
| Eligibility criteria | 6 | Specify study characteristics (e.g., PICOS, length of follow-up) and report characteristics (e.g., years considered, language, publication status) used as criteria for eligibility, giving rationale. | 8 |
| Information sources | 7 | Describe all information sources (e.g., databases with dates of coverage, contact with study authors to identify additional studies) in the search and date last searched. | 7-8 |
| Search | 8 | Present full electronic search strategy for at least one database, including any limits used, such that it could be repeated. | 7-8 |
| Study selection | 9 | State the process for selecting studies (i.e., screening, eligibility, included in systematic review, and, if applicable, included in the meta-analysis). | 9 |
| Data collection process | 10 | Describe method of data extraction from reports (e.g., piloted forms, independently, in duplicate) and any processes for obtaining and confirming data from investigators. | 9 |
| Data items | 11 | List and define all variables for which data were sought (e.g., PICOS, funding sources) and any assumptions and simplifications made. | 10 |
| Risk of bias in individual studies | 12 | Describe methods used for assessing risk of bias of individual studies (including specification of whether this was done at the study or outcome level), and how this information is to be used in any data synthesis. | 10 |
| Summary measures | 13 | State the principal summary measures (e.g., risk ratio, difference in means). | 11 |
| Synthesis of results | 14 | Describe the methods of handling data and combining results of studies, if done, including measures of consistency (e.g., I^2^) for each meta-analysis. | 11-12 |

| **Section/topic** | **#** | **Checklist item** | **Reported on page #** |
| --- | --- | --- | --- |
| Risk of bias across studies | 15 | Specify any assessment of risk of bias that may affect the cumulative evidence (e.g., publication bias, selective reporting within studies). | 12 |
| Additional analyses | 16 | Describe methods of additional analyses (e.g., sensitivity or subgroup analyses, meta-regression), if done, indicating which were pre-specified. | 12 |
| **RESULTS** | | |  |
| Study selection | 17 | Give numbers of studies screened, assessed for eligibility, and included in the review, with reasons for exclusions at each stage, ideally with a flow diagram. | 12 |
| Study characteristics | 18 | For each study, present characteristics for which data were extracted (e.g., study size, PICOS, follow-up period) and provide the citations. | 15 |
| Risk of bias within studies | 19 | Present data on risk of bias of each study and, if available, any outcome level assessment (see item 12). | 16 |
| Results of individual studies | 20 | For all outcomes considered (benefits or harms), present, for each study: (a) simple summary data for each intervention group (b) effect estimates and confidence intervals, ideally with a forest plot. | 18 |
| Synthesis of results | 21 | Present results of each meta-analysis done, including confidence intervals and measures of consistency. | 18-19 |
| Risk of bias across studies | 22 | Present results of any assessment of risk of bias across studies (see Item 15). | 20 |
| Additional analysis | 23 | Give results of additional analyses, if done (e.g., sensitivity or subgroup analyses, meta-regression [see Item 16]). | 21 |
| **DISCUSSION** | | |  |
| Summary of evidence | 24 | Summarize the main findings including the strength of evidence for each main outcome; consider their relevance to key groups (e.g., healthcare providers, users, and policy makers). | 21 |
| Limitations | 25 | Discuss limitations at study and outcome level (e.g., risk of bias), and at review-level (e.g., incomplete retrieval of identified research, reporting bias). | 25 |
| Conclusions | 26 | Provide a general interpretation of the results in the context of other evidence, and implications for future research. | 25 |
| **FUNDING** | | |  |
| Funding | 27 | Describe sources of funding for the systematic review and other support (e.g., supply of data); role of funders for the systematic review. | 26 |

**eMethods 2. Search strategy for EMBASE**

Database: Embase Classic+Embase <1947 to 2021 September 08>

Search Strategy:

--------------------------------------------------------------------------------

1 Ambulances.mp. [mp=title, abstract, heading word, drug trade name, original title, device manufacturer, drug manufacturer, device trade name, keyword, floating subheading word, candidate term word] (2759)

2 Emergency Medical Technicians.mp. [mp=title, abstract, heading word, drug trade name, original title, device manufacturer, drug manufacturer, device trade name, keyword, floating subheading word, candidate term word] (1214)

3 Air Ambulances/ (2928)

4 Emergency Medical Services.mp. [mp=title, abstract, heading word, drug trade name, original title, device manufacturer, drug manufacturer, device trade name, keyword, floating subheading word, candidate term word] (12512)

5 paramedic*.tw. (13167)

6 ems.tw. (19351)

7 emt.tw. (37368)

8 prehospital.tw. (18005)

9 pre-hospital.tw. (8679)

10 first responder*.tw. (3358)

11 emergency medical technicians.tw. (1071)

12 emergency services.tw. (5913)

13 ambulance*.tw. (17448)

14 HEMS.tw. (1097)

15 out-of-hospital.tw. (19583)

16 field triage.tw. (379)

17 1 or 2 or 3 or 4 or 5 or 6 or 7 or 8 or 9 or 10 or 11 or 12 or 13 or 14 or 15 or 16 (125445)

18 stress disorders, traumatic/ or psychological trauma/ or stress disorders, post-traumatic/ or stress disorders, traumatic, acute/ or stress, psychological/ or re experienc*.mp. or traumatic neuros*.mp. or traumatic stress.mp. or posttraumatic*.mp. or post traumatic*.mp. or stress disorder*.mp. or acute stress.mp. or ptsd.mp. or asd.mp. or desnos.mp. or extreme stress.mp. or flashback*.mp. or flash back*.mp. or psych* stress.mp. or psych* trauma*.mp. or psycho?traumv.tw. [mp=title, abstract, heading word, drug trade name, original title, device manufacturer, drug manufacturer, device trade name, keyword, floating subheading word, candidate term word] (265644)

19 trauma*.mp. and (avoidance or grief or horror or death* or nightmare* or night mare* or emotion*).tw. [mp=title, abstract, heading word, drug trade name, original title, device manufacturer, drug manufacturer, device trade name, keyword, floating subheading word, candidate term word] (64454)

20 burnout, professional/ (1513)

21 (fatigue* or burnout* or burntout* or "burn* out*").tw. (194317)

22 Depression/ (404582)

23 depress*.tw. (699689)

24 Depressive disorders.mp. [mp=title, abstract, heading word, drug trade name, original title, device manufacturer, drug manufacturer, device trade name, keyword, floating subheading word, candidate term word] (15199)

25 depressive disorders, major.mp. [mp=title, abstract, heading word, drug trade name, original title, device manufacturer, drug manufacturer, device trade name, keyword, floating subheading word, candidate term word] (38)

26 Anxiety.mp. [mp=title, abstract, heading word, drug trade name, original title, device manufacturer, drug manufacturer, device trade name, keyword, floating subheading word, candidate term word] (421976)

27 Anxiety Disorders.mp. [mp=title, abstract, heading word, drug trade name, original title, device manufacturer, drug manufacturer, device trade name, keyword, floating subheading word, candidate term word] (31717)

28 (anxiety or anxious).tw. (323018)

29 mood disorders.mp. [mp=title, abstract, heading word, drug trade name, original title, device manufacturer, drug manufacturer, device trade name, keyword, floating subheading word, candidate term word] (24127)

30 Mental Disorders.mp. [mp=title, abstract, heading word, drug trade name, original title, device manufacturer, drug manufacturer, device trade name, keyword, floating subheading word, candidate term word] (72936)

31 distress*.tw. (199135)

32 18 or 19 or 20 or 21 or 22 or 23 or 24 or 25 or 26 or 27 or 28 or 29 or 30 or 31 (1621178)

33 (treat* or psychotherap* or psycho therap* or talk* therap* or therapeutic technique* or therapist* or third wave).mp. or time limited.tw. [mp=title, abstract, heading word, drug trade name, original title, device manufacturer, drug manufacturer, device trade name, keyword, floating subheading word, candidate term word] (9572962)

34 (((behaviour* or behavior*) adj2 cognitiv*) or cbt or ccbt or ((behav* or cognitive*) adj3 (intervention* or manag* or program* or restructure* or therap* or treat*)) or (stress inoculation adj2 (intervention* or program* or therap* or train* or treat*)) or (behav* adj2 activat*) or ((trauma adj (based or focused or led)) or exposure based or prolonged exposure)).tw. (184522)

35 emotion focused therapy/ or sympathy.mp. [mp=title, abstract, heading word, drug trade name, original title, device manufacturer, drug manufacturer, device trade name, keyword, floating subheading word, candidate term word] (1973)

36 (((compassion or emotion* or emotive*) adj (based or focused or led)) or emotional processing or ((compassion or emotion* or emotive*) adj3 (coach* or intervention* or program* or therap* or treat*))).ti,ab. (15306)

37 exposure therapy/ or narrative therapy/ or virtual reality exposure therapy.mp. [mp=title, abstract, heading word, drug trade name, original title, device manufacturer, drug manufacturer, device trade name, keyword, floating subheading word, candidate term word] (2263)

38 (((augmented or virtual) adj2 reality) or (virtual adj (environment or restorative)) or ((exposure or implosive or virtual reality) adj2 (intervention* or program* or therap* or train*))).ti,ab. (28032)

39 ((imagery adj2 (rehears* or re hears*)) or (((lower* or reduc*) adj3 (bad dream* or nightmare*)) and (intervention* or program* or therap* or treat*)) or ((intervention* or program* or therap* or treat*) adj3 nightmare*)).mp. or ((presleep or presleep) adj2 imagery).ti,ab. (521)

40 "debriefing (psychological)".mp. [mp=title, abstract, heading word, drug trade name, original title, device manufacturer, drug manufacturer, device trade name, keyword, floating subheading word, candidate term word] (2)

41 eye movement desensitization reprocessing.mp. [mp=title, abstract, heading word, drug trade name, original title, device manufacturer, drug manufacturer, device trade name, keyword, floating subheading word, candidate term word] (54)

42 Psychotherapy.mp. [mp=title, abstract, heading word, drug trade name, original title, device manufacturer, drug manufacturer, device trade name, keyword, floating subheading word, candidate term word] (118392)

43 behavior therapy.mp. [mp=title, abstract, heading word, drug trade name, original title, device manufacturer, drug manufacturer, device trade name, keyword, floating subheading word, candidate term word] (50247)

44 (behavi?r adj3 therap*).tw. (9655)

45 cognitive therapy.mp. [mp=title, abstract, heading word, drug trade name, original title, device manufacturer, drug manufacturer, device trade name, keyword, floating subheading word, candidate term word] (44939)

46 (cognitive adj3 therap*).tw. (35263)

47 (relax* adj3 (technique* or therap*)).tw. (5677)

48 exp mind body therapies/ (62461)

49 guided imagery.tw. (1132)

50 "imagery (Psychotherapy)"/ (1490)

51 PSYCHOTHERAPY, GROUP.mp. [mp=title, abstract, heading word, drug trade name, original title, device manufacturer, drug manufacturer, device trade name, keyword, floating subheading word, candidate term word] (603)

52 ((psychoeducation or psycho-education) adj2 group*).tw. (597)

53 (group* adj3 (therap* or program*)).tw. (74234)

54 mindfulness.tw. (11859)

55 33 or 34 or 35 or 36 or 37 or 38 or 39 or 40 or 41 or 42 or 43 or 44 or 45 or 46 or 47 or 48 or 49 or 50 or 51 or 52 or 53 or 54 (9746068)

56 17 and 32 and 55 (3112)

***************************

**eFigure 1.** *Ratings of studies included in the review on the 9 EPOC risk of bias criteria*


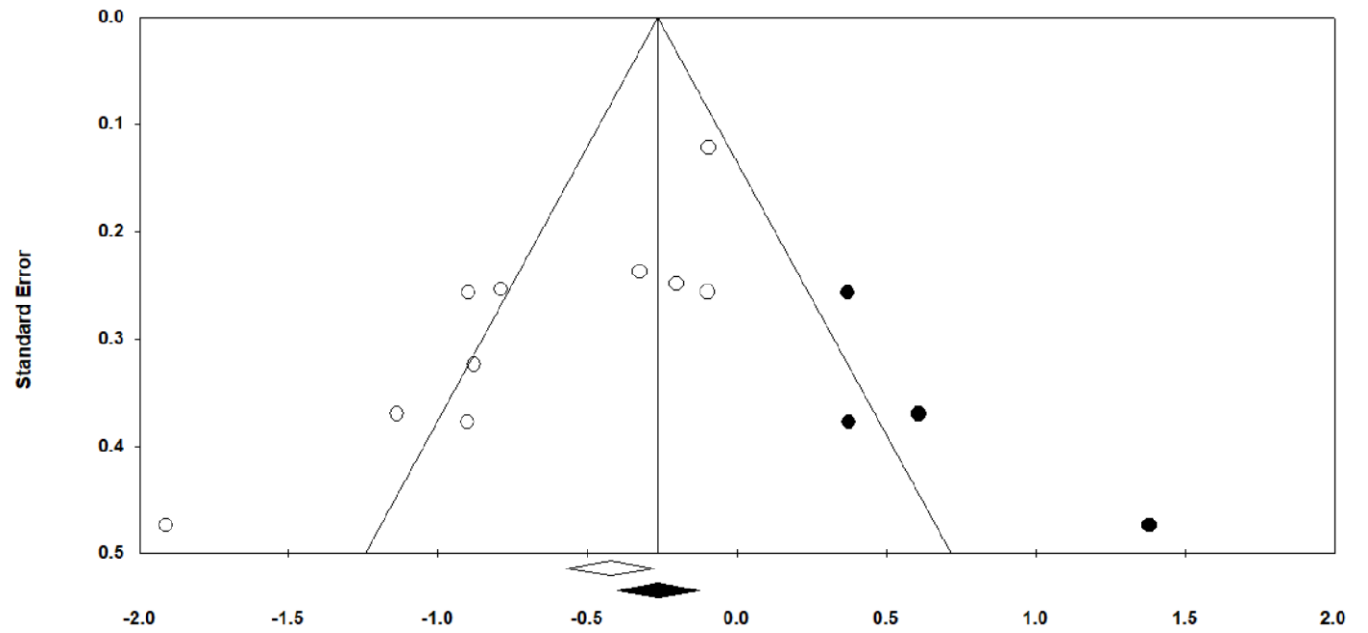
**eFigure 2.** *Funnel plot of standard differences of mean versus standard errors for depression*

***
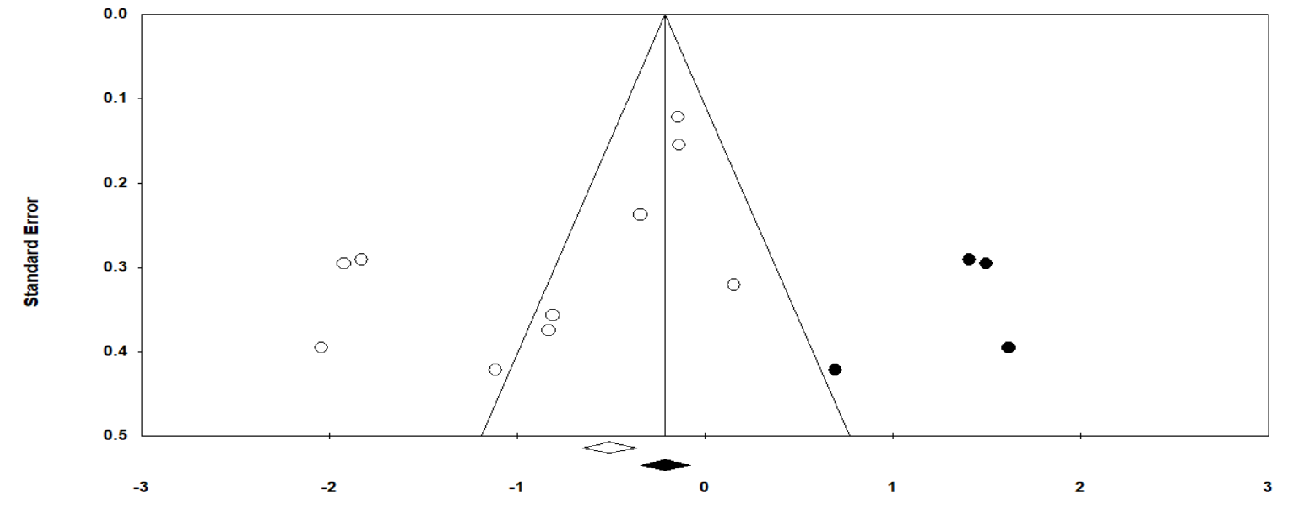
*eFigure 3.** *Funnel plot of standard differences of mean versus standard errors for PTSD*

**eFigure 4.** *Funnel plot of standard differences of mean versus standard errors for anxiety*


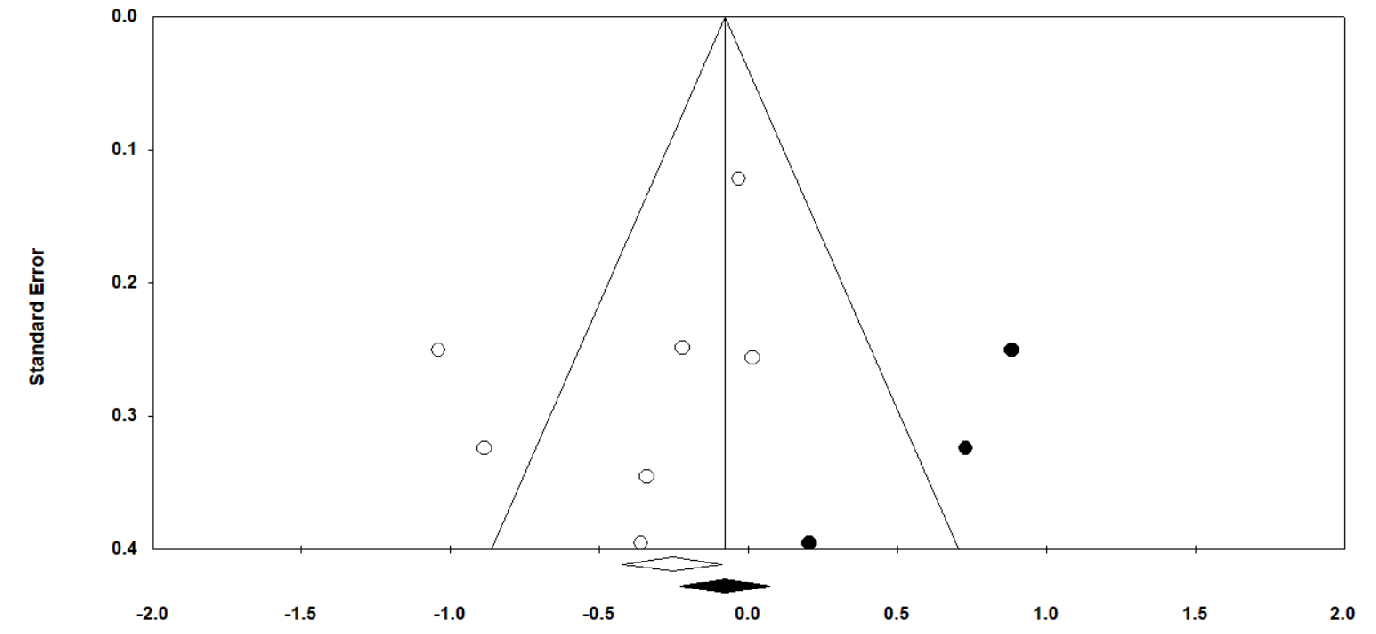


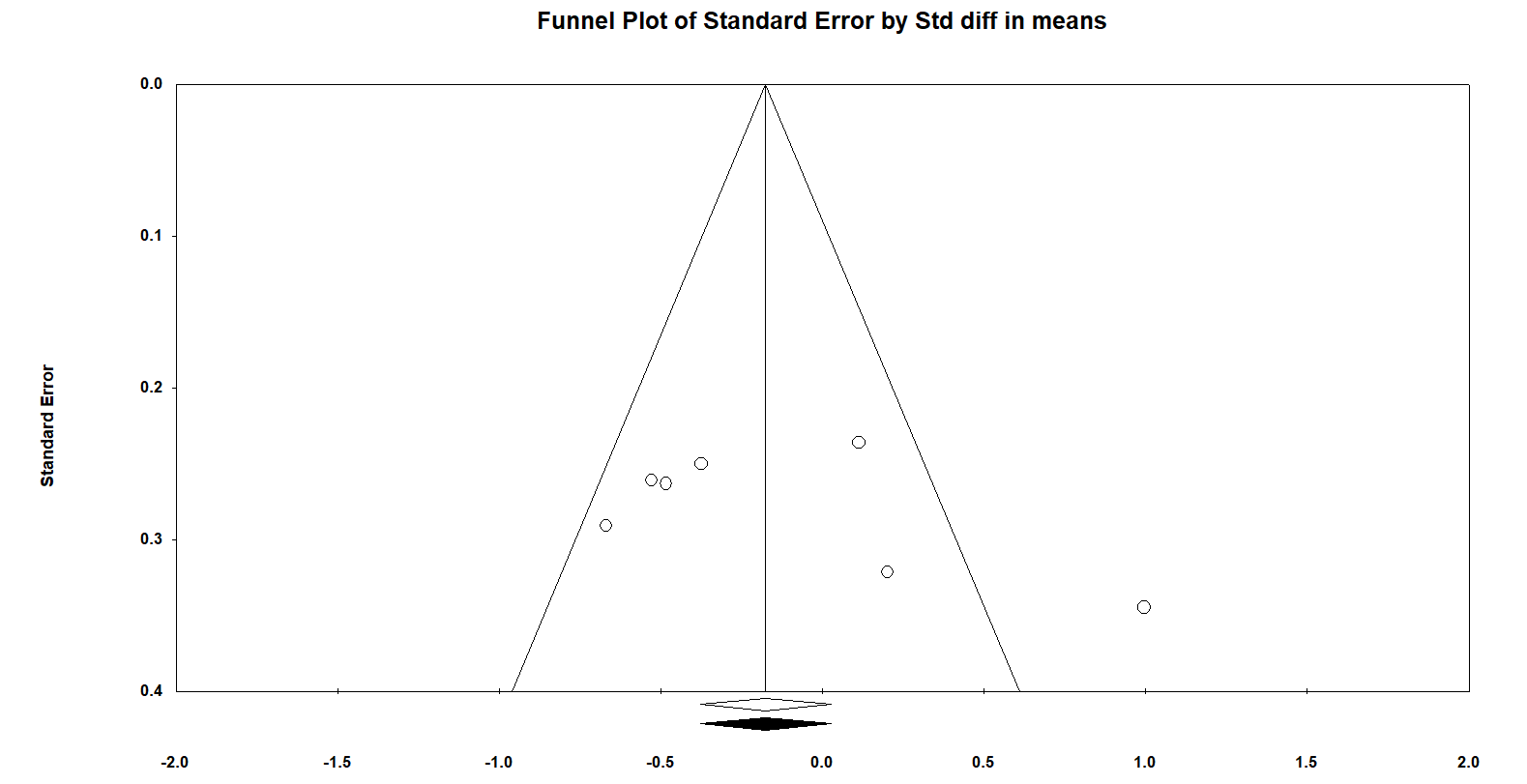
**eFigure 5.** *Funnel plot of standard differences of mean versus standard errors for stress*
